# Inflammatory Phenotype of Intrahepatic Sulfatide-Reactive Type II NKT Cells in Humans With Autoimmune Hepatitis

**DOI:** 10.3389/fimmu.2019.01065

**Published:** 2019-05-28

**Authors:** Marcial Sebode, Jennifer Wigger, Pamela Filpe, Lutz Fischer, Sören Weidemann, Till Krech, Christina Weiler-Normann, Moritz Peiseler, Johannes Hartl, Eva Tolosa, Johannes Herkel, Christoph Schramm, Ansgar W. Lohse, Philomena Arrenberg

**Affiliations:** ^1^Department of Medicine, University Medical Center Hamburg-Eppendorf, Hamburg, Germany; ^2^European Reference Network on Hepatological Diseases (ERN RARE-LIVER), Hamburg, Germany; ^3^Department of Hepatobiliary Surgery and Transplantation, University Medical Center Hamburg-Eppendorf, Hamburg, Germany; ^4^Department of Pathology, University Medical Center Hamburg-Eppendorf, Hamburg, Germany; ^5^Martin Zeitz Center for Rare Diseases, University Medical Center Hamburg-Eppendorf, Hamburg, Germany; ^6^Institute of Immunology, University Medical Center Hamburg-Eppendorf, Hamburg, Germany

**Keywords:** alpha-galactosylceramide, CD1d, lipid antigen, tumor necrosis factor-alpha, unconventional T cells

## Abstract

**Background:** Natural Killer T (NKT) cells are CD1d-restricted innate-like T cells that can rapidly release stored cytokines upon recognition of lipid antigens. In mice, type I NKT cells seem to promote liver inflammation, whereas type II NKT cells seem to restrict hepatitis. Here, we aimed at characterizing the role of human type I and type II NKT in patients with autoimmune hepatitis (AIH).

**Methods:** NKT cells were analyzed by flow cytometry in peripheral blood and liver of AIH patients and control groups. α-galactosylceramide-loaded or sulfatide-loaded tetramers were used to detect type I or II NKT cells, respectively. Hepatic CD1d was stained by *in situ*-hybridization of liver biopsies.

**Results and Conclusions:** Type II NKT cells were more prevalent in human peripheral blood and liver than type I NKT cells. In AIH patients, the frequency of sulfatide-reactive type II NKT cells was significantly increased in peripheral blood (0.11% of peripheral blood leukocytes) and liver (3.78% of intrahepatic leukocytes) compared to healthy individuals (0.05% and 1.82%) and patients with drug-induced liver injury (0.06% and 2.03%; *p* < 0.05). Intrahepatic type II NKT cells of AIH patients had a different cytokine profile than healthy subjects with an increased frequency of TNFα (77.8% vs. 59.1%, *p* < 0.05), decreased IFNγ (32.7% vs. 63.0%, *p* < 0.05) and a complete lack of IL-4 expressing cells (0% vs. 2.1%, *p* < 0.05). T cells in portal tracts expressed significantly more CD1d-RNA in AIH livers compared to controls. This study supports that in contrast to their assumed protective role in mice, human intrahepatic, sulfatide-reactive type II NKT cells displayed a proinflammatory cytokine profile in patients with AIH. Infiltrating T cells in portal areas of AIH patients overexpressed CD1d and could thereby activate type II NKT cells.

## Introduction

Autoimmune hepatitis (AIH) is a chronic inflammatory liver disease with unknown pathogenesis (1). Clinical features of AIH comprise elevation of transaminases and IgG/gammaglobulins, the presence of autoantibodies like ANA, anti-SMA, anti-SLA/LP, and anti-LKM and lymphoplasmacellular infiltrates on liver histology (2). The association of AIH with specific human leukocyte antigens (HLA) suggests a role of the adaptive immune system in promoting an antigen-driven immune response (3). However, in most cases of AIH, the liver-specific antigen driving the chronic inflammation is unknown. Currently, standard treatment for AIH consists of unspecific immunosuppression with predniso(lo)ne and azathioprine (2). To offer AIH patients a more selective treatment in the future, it is essential to identify disease-specific mediators of intrahepatic inflammation.

NKT cells exhibit both Natural Killer (NK) cell markers (like CD161 or CD56) and a T cell receptor (TCR) recognizing lipid antigens as well as endogenous hydrophobic peptides when presented by the MHC class I-like molecule CD1d (4). Due to their unconventional antigen recognition and their restricted TCR expression patterns, they belong to the group of unconventional T cells. Upon stimulation of their TCR, NKT cells can rapidly secrete stored cytokines and thus orchestrate effector functions. So far, NKT cells have been mainly characterized in mouse models and have been subdivided into pro-inflammatory type I NKT cells and presumably protective type II NKT cells with immunoregulatory potential (5–8).

Type I NKT cells (invariant NKT cells, iNKT) possess a semi-invariant TCR (encoded by the Vα14-Jα18 gene segments in mice and Vα24-Jα18 in humans) recognizing predominantly α-linked glycolipids such as the ligand α-galactosylceramide (αGalCer). In addition, several natural ligands have been identified including bacterial wall components and the murine self-antigen isoglobotrihexosyl ceramide (9, 10). Type I NKT cells are abundant in murine liver, but represent only a small population of human intrahepatic immune cells (11). They can be detected specifically by flow cytometry using αGalCer-loaded CD1d-tetramers (12).

Type II NKT cells are more heterogeneous and exhibit oligoclonal TCRs (13). They recognize the glycosphingolipid 3′-sulfated β-galactosylceramide (called sulfatide), which is part of plasma membranes in different tissues such as hepatocytes, pancreatic β-cells, kidneys, and myelin sheaths, and thus could serve as self-antigen (14–17). Type II NKT cells have been identified specifically in mice by using sulfatide-loaded murine CD1d-tetramers (15). Recently, it has been suggested that type II NKT cells are also reactive to phospholipids and to other glycosphingolipids such as β-glucosyl ceramide 22:0 (βGL1-22) and glucosyl sphingosine (LGL1) (18–21).

The functions of NKT cells in inflammatory liver disease have been mainly investigated in mice. In Concanavalin A (Con A)-induced hepatitis, a murine model of AIH, activated sulfatide-reactive type II NKT cells exert a regulatory function on pathogenic type I NKT cells by inducing anergy in type I NKT cells and thus mediating protection from liver injury (5). Consistently, sulfatide-mediated activation of type II NKT cells inhibited type I NKT cell-induced inflammation in hepatic ischemic reperfusion injury and alcoholic liver disease in mice (6, 22). In addition to their interaction with type I NKT cells, type II NKT cells can also interact with conventional T cells and thereby induce spontaneous, chronic liver inflammation in a mouse model resembling AIH (23). In human AIH, CD3+CD56+ NKT cells seemed to be diminished in peripheral blood in active disease and produced less IL-4 (24). Consistently, a small study found mRNA expression of Vα24 (as indicator for type I NKT cells) reduced in peripheral blood and augmented in livers of children with AIH (25). These results hint at both type I and type II NKT cells taking part in the pathogeneses of immune-mediated liver diseases. However, specific tetramer-based analyses for type I and type II NKT cells in human AIH are missing.

In this study, we addressed which role NKT cells play in human AIH. Thus, we have analyzed type I and type II NKT cells in peripheral blood and liver samples of AIH patients in comparison to patients with drug-induced liver injury (DILI) and healthy subjects. To verify the specificity of our results for NKT cells, other unconventional T cells, such as Mucosal-Associated Invariant T (MAIT) cells and gamma-delta (γδ) T cells, were also analyzed in AIH patients and control groups. We report a significant increase of intrahepatic sulfatide-reactive type II NKT cells only in AIH patients. Moreover, CD1d was up-regulated on infiltrating portal T cells in AIH. This was associated with increased production of tumor necrosis factor-alpha (TNFα), decreased production of interferon gamma (IFNγ) and a complete lack of IL-4 by intrahepatic sulfatide-reactive type II NKT cells, revealing their unexpected pro-inflammatory potential in human AIH.

## Methods

### Human Subjects

A total of 74 consecutive patients with AIH (46 in whom blood samples and 28 in whom liver biopsies were analyzed) and 21 DILI patients (10 in whom blood samples and 11 in whom liver biopsies were analyzed), who attended the YAEL-Center for Autoimmune Liver Diseases, I. Department of Medicine of the University Medical Center Hamburg-Eppendorf were included in the study. Diagnosis of AIH was based on international guidelines and diagnosis of DILI was due to the RUCAM score (2, 26–28). DILI patients were followed-up for at least 1 year after acute onset of hepatitis thereby assuring that liver enzymes did not rise again after withdrawal of the causative drug. Liver biopsies for experimental analyses were taken during mini-laparoscopy and only performed when indicated for clinical reasons. Additionally, 14 patients with liver adenomas, two with hepatic metastases of neuroendocrine tumors and one with fibrolamellar hepatocellular carcinoma who underwent surgery in the Department of Hepatobiliary Surgery and Transplantation of the University Medical Center Hamburg-Eppendorf, served for analyses of healthy liver in the resection margin. In 39 healthy control subjects, peripheral blood was analyzed. Clinical characteristics of patients that served for analyses out of peripheral blood or liver biopsies are shown in Tables 1 or 2, respectively. Additional clinical information is presented in the Supplementary Material. All patients gave their written informed consent. The study was approved by the local ethics committee (PV3912, PV5184, and PV5187) and the study protocol conformed to the ethical guidelines of the 1975 Declaration of Helsinki.

**Table 1:** Clinical characteristics of AIH patients and control groups at the time of analyses of peripheral unconventional T cells.

**Study group**	**Age (years)**	**Sex (f/m)**	**ALT (U/l)**	**AST (U/l)**	**ALP (U/l)**	**Total bilirubin (mg/dl)**	**IgG (g/l)**	**Liver cirrhosis**	**Treatmentnaive**
AIH all	47	35/11	42	38	80	0.7	14.9	10/46	13/46
(*n* = 46)	(22–77)		(11–2609)	(13–2359)	(33–367)	(0.2–16.4)	(8.6–36.4)		
AIH naive	37	9/4	476	424	105	1.0	18.3	0/13	13/13
(*n* = 13)	(25–27)		(54–2609)	(30–2359)	(44–367)	(0.4–16.4)	(9.7–33.0)		
AIH treated	53	26/7	34	33	76	0.6	13.6	10/33	0/33
(*n* = 33)	(22–77)		(11–1593)	(13–558)	(33–240)	(0.2–1.6)	(7.1–36.4)		
Healthy	33	16/23	N/A	N/A	N/A	N/A	N/A	N/A	N/A
(*n* = 39)	(19–41)								
DILI	53	8/2	1172	775	126	1.9	11.8	0/10	10/10
(*n* = 10)	(33–60)								
			(17–3055)	(22–1623)	(53–230)	(0.2–23.3)	(7.7–18.4)		

*AIH, autoimmune hepatitis; ALP, alkaline phosphatase; ALT, alanine aminotransferase; AST, aspartate aminotransferase; DILI, drug-induced liver injury; IgG, immunoglobulin G; mHAI, modified Hepatic Activity Index; N/A, not available. Normal values: ALT and AST <35U/l (female), <50U/l (male); ALP <104U/l (female), <129U/l (male); total bilirubin <1.2 mg/dl; IgG 7–16g/l. Median values and range are given*.

**Table 2:** Clinical characteristics of AIH patients and control groups at the time of analyses of intrahepatic unconventional T cells (at the time of liver biopsies).

**Study group**	**Age (years)**	**Sex (f/m)**	**ALT (U/l)**	**Total bilirubin (mg/dl)**	**IgG (g/l)**	**mHAI**	**Fibrosis**	**Liver cirrhosis**	**Treatment naive**
AIH all	53	20/8	247	1.0	18.9	7	2	4/28	16/28
(*n* = 28)	(24–78)		(11–2609)	(0.4–16.4)	(7.6–36.4)	(4–14)	(0–4)		
AIH naive	51	10/6	776	2.2	17.3	8	1	2/16	16/16
(*n* = 16)	(25–77)		(58–2609)	(0.5–16.4)	(9.7–33.0)	(4–14)	(0–4)		
AIH treated	55	10/2	118	0.4	19.4	7	2	2/16	0/12
(*n* = 12)	(24–78)		(11–1593)	(0.4–1.5)	(7.6–36.4)	(4–10)	(0–4)		
Healthy	56.5	10/7	30	0.5	N/A	N/A	0	0/17	14/17
(*n* = 17)	(18–73)		(16–47)	(0.3–0.8)			(0–0)		
DILI	57	9/2	1228	1.2	11.2	6	0	0/11	11/11
(*n* = 11)	(28–68)		(37–3055)	(0.2–23.3)	(7.9–18.4)	(1–10)	(0–1)		

*AIH, autoimmune hepatitis; ALT, alanine aminotransferase; DILI, drug-induced liver injury; IgG, immunoglobulin G; mHAI, modified Hepatic Activity Index; N/A, not available. Normal values: ALT <35U/l (female), <50U/l (male); total bilirubin <1.2 mg/dl; IgG 7-16g/l. Range of histological scores: mHAI 0-18; Fibrosis 0 (no fibrosis)-4 (liver cirrhosis). Median values and range are given*.

### Liver Histology

The modified hepatic activity index (mHAI) score has been established for assessing the histological activity of chronic active hepatitis and is recommended for the quantification of intrahepatic inflammatory activity of AIH (2, 29). We have applied the mHAI score on our cases of acute manifestation of AIH (for which the mHAI score has not been validated yet) and on DILI cases (since there exists no recommended histological score to quantify the inflammatory activity of immune-mediated DILI). However, we found it feasible to apply the mHAI score on these cases as an approach to compare histological inflammation of acute AIH and DILI. Histopathological reports confirmed that the resection margins of liver tumors were free of inflammation, fibrosis or steatosis. Histological assessment was performed by pathologists of our Department of Pathology (SW, TK).

### Flow Cytometry of Peripheral Blood or Intrahepatic Unconventional T Cells

PBMC were isolated from peripheral blood of patients and healthy subjects as previously described (30). PBMC were stained with various fluorochrome-conjugated antibodies (FITC anti-human CD3, Clone OKT3; PE/Cy7 anti-human CD3, clone SK7; Pacific Blue™ anti-human CD4, clone RPA-T4; PerCP/Cy5.5 anti-human CD4, clone RPA-T4; APC anti-human CD8a, clone RPA-T8; APC anti-human CD14, clone HCD14; PE/Cy5 anti-human CD19, clone HIB19; PE/Cy7 anti-human CD19, clone HIB19; PerCP/Cy5.5 anti-human CD45, clone HI30; PE/Dazzle™ 594 anti-human CD183 (CXCR3), clone G025H7; APC anti-human CD184 (CXCR4), clone 12G5; PerCP/Cy5.5 anti-human CD186 (CXCR6), clone K041E5; PE/Cy7 anti-human CD199 (CCR9), clone L053E8; APC/Cy7 anti-human IFN-γ, clone 4S.B3; APC anti-human IL-4, clone MP4-25D2; FITC anti-human IL-17A, clone BL168; FITC anti-human TCR Vα7.2, clone 3C10; APC anti-human TNF-α, clone MAb11, all Biolegend, Koblenz, Germany; Alexa Fluor® 700 anti-human CD8, clone RPA-T8; Alexa Fluor® 700 anti-human CD14, clone M5E2; FITC anti-Human TCRγ/δ, clone 11F2, all BD Biosciences, Heidelberg, Germany. NKT cells were stained with lipid-loaded (α-Galactosyl Ceramide, Avanti Polar Lipids, Alabaster, Alabama, USA; or Sulfatides, Matreya, State College, Pennsylvania, USA) fluorochrome-labeled tetramers (kindly provided by the NIH Tetramer Core Facility, Atlanta, Georgia, USA). Liver biopsies were strained through a 100 μm mesh (EASYstrainer™, 100 μm mesh size, Greiner bio one, Frickenhausen, Germany), and cells obtained were washed several times before antibody staining. Flow cytometry analyses were performed with BD LSR II (BD Bioscienes, Heidelberg, Germany). For further details of flow cytometric analyses or lipid-loading of tetramers, we refer to the Supplementary Material.

### Real-Time Quantitative PCR Analysis of CD1d in Liver Tissue

RNA was isolated from whole liver tissue samples from AIH or DILI liver biopsies or healthy liver margin of resected liver adenomas using an RNA Isolation kit (NucleoSpin® RNA, Macherey-Nagel, Düren, Germany). For reverse transcription the High Capacity cDNA Reverse Transcription kit (Applied biosystems, Darmstadt, Germany) was used according to the manufacturer's protocol. Amplification of CD1d-cDNA (CD1dTaqMan® Gene Expression, sequence Hs00939886_g1, ThermoFisher Scientific, Waltham, Massachusetts, USA) was performed with KAPA PROBE FAST qPCR Master Mix (KAPA Biosystems, Wilmington, Delaware, USA) and ROX fluorescein reference dyes (KAPA Biosystems, Wilmington, Delaware, USA). After initial denaturation for 20 s at 95°C, cycles of primer annealing at 60°C for 20 s and elongation at 95°C for 1 s were followed. Forty cycles of amplification steps were applied. Mean relative expression of CD1d was normalized to HPRT1 (HPRT1TaqMan® Gene Expression, sequence Hs02800695_m1, ThermoFisher Scientific, Waltham, Massachusetts, USA) and calculated using the 2^−ΔΔ*CT*^ method.

### Immunohistochemistry of CD1d in Liver Biopsies

CD1d-mRNA was stained in liver biopsies of AIH and DILI patients. Co-staining with anti-CK7 or anti-CK18 antibodies was performed to detect CD1d expression in cholangiocytes or hepatocytes. Five micrometer-sections of formalin-fixed paraffine embedded (FFPE) tissue slides were deparaffinized by using xylene and ethanol after incubation at 60°C. Tissue sections were blocked with H_2_O_2_ and were boiled in target retrieval solution. The CD1d target probe (RNAscope® Probe- Hs-CD1D-O1, sequence 1374–2626 of NM_001766.3, Acd Bio, Milano, Italy) was applied (120 min at 40°C) after protease treatment and amplifier solutions were used for the amplification steps (15–30 min at 40°C). Then, tissue sections were incubated with a peroxidase-labeled probe and detection was followed by the usage of DAB (DAB Substrate Kit, Abcam, Cambridge, UK). Specific staining of CD1d was assured by comparison with positive (RNAscope® Positive Control Probe- Hs-PPIB, sequence 139-989 of NM_000942.4, Acd Bio, Milano, Italy) and negative controls (RNAscope® Negative Control Probe- DapB, sequence 414–862 of EF191515, Acd Bio, Milano, Italy). For immunohistochemical co-stainings, tissue slides were first incubated in normal sera (1 h at room temperature) and after that with antibodies (anti-CD4, Abcam, Cambridge, UK) anti-CD8 (Abcam, Cambridge, UK), anti-Cytokeratin 7 (anti-CK7, clone RCK105, Abcam, Cambridge, UK) or anti-Cytokeratin 18 HRP (anti-CK18, clone DC-10, Santa Cruz, Heidelberg, Germany) overnight at 4°C. Then, tissue sections with anti-CK7, anti-CK18, anti-CD4 or anti-CD8 stainings were incubated with a secondary antibody (anti-mouse, goat anti-mouse IgG H&L (HRP), Abcam, Cambridge, UK or anti-rabbit HRP, Abcam, Cambridge, UK for 1 h at room temperature). Immunohistochemical stainings were detected by DAB and slides were counterstained with hematoxylin. For quantification of CD1d-expressing cells, five representative high-power fields (60-fold magnification) of lobular or portal areas of a liver biopsy were analyzed in a blinded manner.

### Statistical Analysis

Statistical analyses were performed using GraphPad Prism software (version 6, Graphpad, Software, San Diego, California, USA). Data were analyzed with the Mann-Whitney *U* test. Median values (horizontal bars) and interquartile range (IQR) are shown. For statistical analysis of three or more groups, we performed one-way ANOVA, Dunn's multiple comparisons test and Tukey's post-test. Differences in means of experimental groups with *p* < 0.05 were considered significant. These formatting styles are meant as a guide, as long as the heading levels are clear, Frontiers style will be applied during typesetting.

## Results

### Selective Increase of Peripheral Blood and Intrahepatic Sulfatide-Reactive Type II NKT Cells in AIH Patients

**Figure 1:**
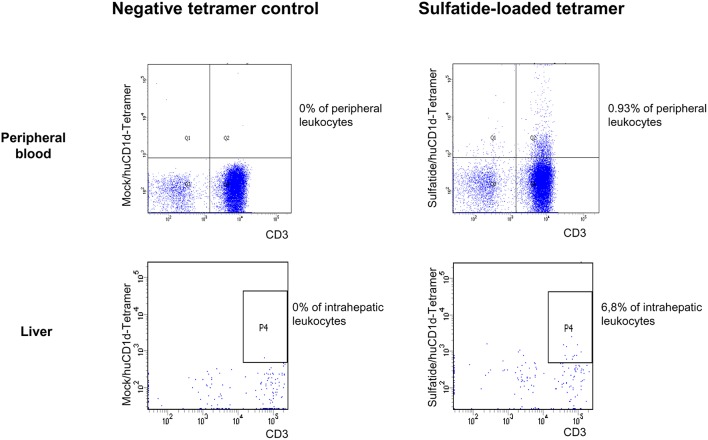
Exemplary CD1d tetramer stainings. Mock-loaded human CD1d tetramers served as negative control staining for lipid-loaded human CD1d tetramers. An exemplary peripheral blood (upper row) and intrahepatic (lower row) flow cytometric staining with mock-loaded human CD1d tetramer (left) and sulfatide-loaded human CD1d tetramer (right) from an AIH patient is shown.

**Figure 2:**
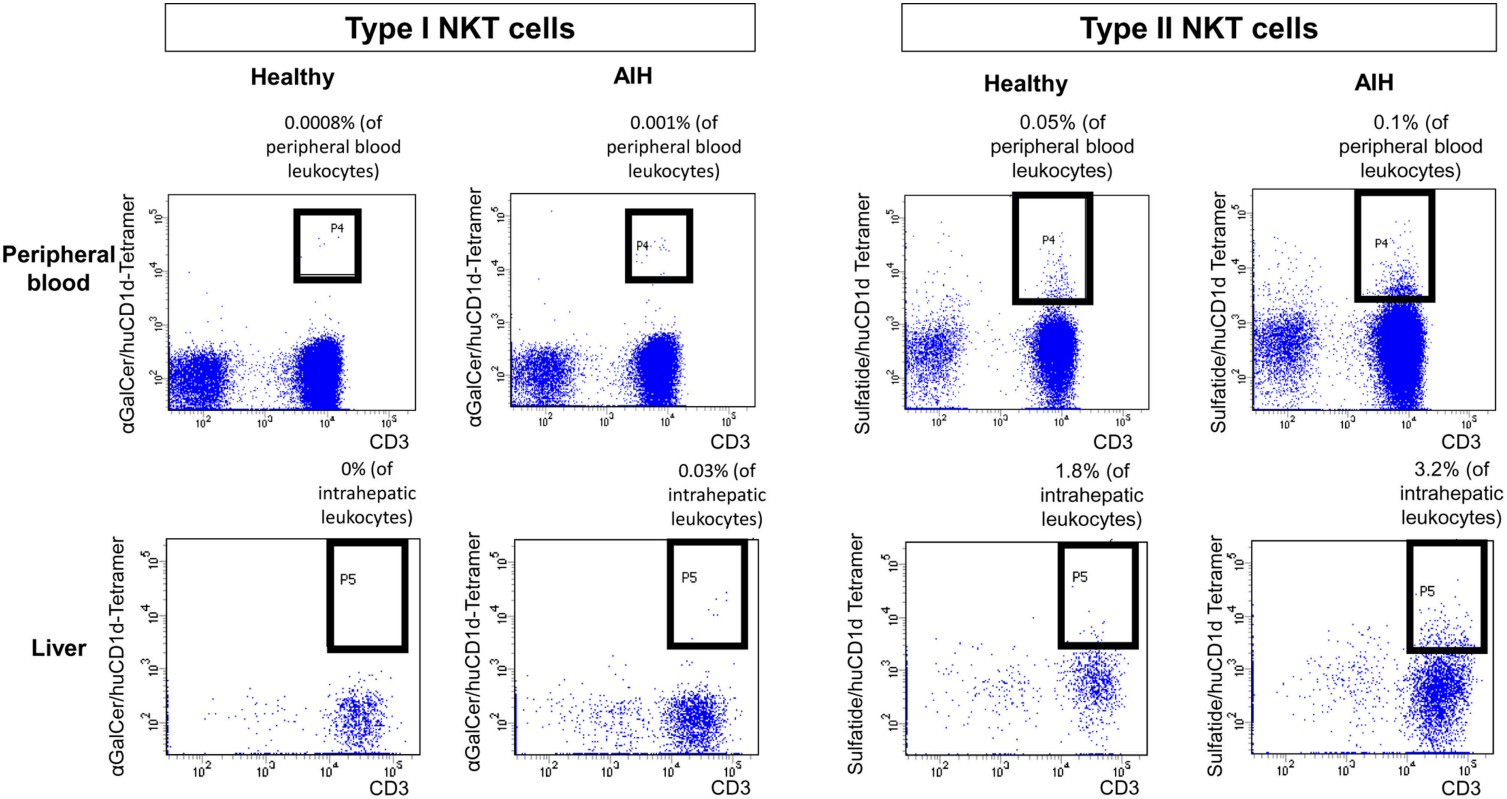
Exemplary flow cytometric stainings of type I and type II NKT cells. Human (hu) CD1d-tetramers loaded either with αGalCer or sulfatide were used to stain type I (left) or type II (right) NKT cells in peripheral blood (upper row) or in liver biopsies (lower row). Representative flow cytometric stainings of type I and type II NKT cells in a healthy subject and a patient with AIH are displayed.

**Figure 3:**
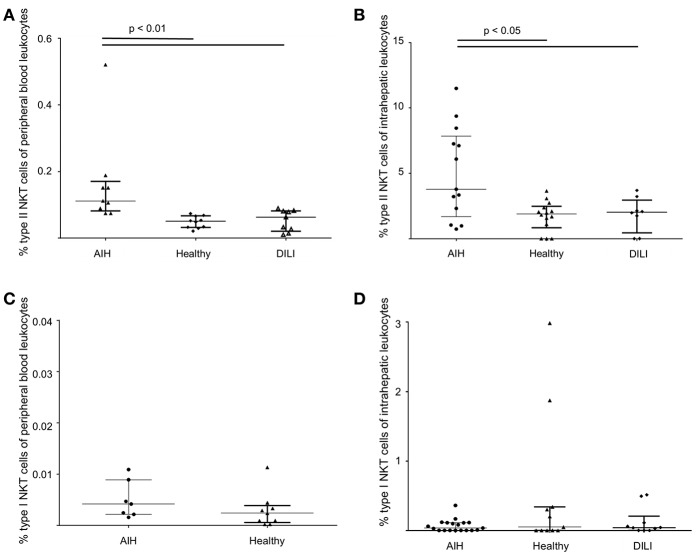
Increased frequency of sulfatide-reactive type II NKT cells in patients with autoimmune hepatitis. The frequency of peripheral blood **(A)** and intrahepatic **(B)** type II NKT cells and peripheral blood **(C)** and intrahepatic **(D)** type I NKT cells in AIH patients in comparison to healthy subjects and patients with DILI is shown.

**Figure 4:**
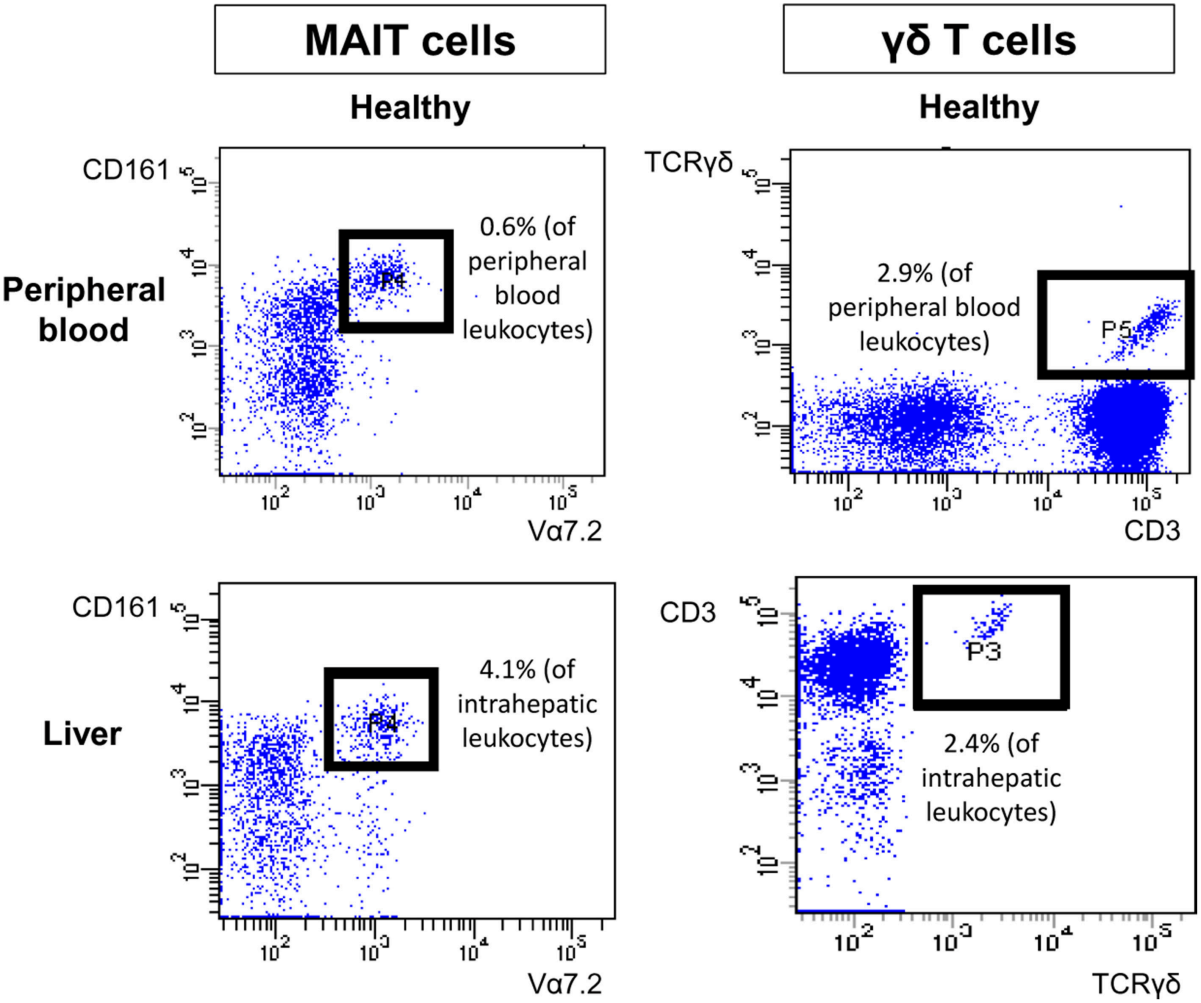
Exemplary flow cytometric stainings of peripheral and intrahepatic MAIT and γδ T cells. Representative flow cytometric stainings of peripheral blood (upper row) and intrahepatic (lower row) MAIT cells (left) and γδ T cells (right) of a healthy individual are shown.

**Figure 5:**
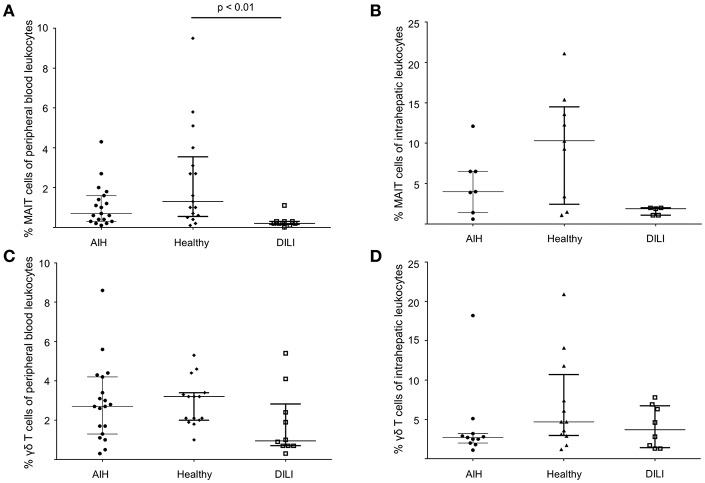
No increased infrequency of peripheral blood and intrahepatic MAIT cells or γδ T cells in AIH patients. To exclude unspecific elevation of type II NKT in AIH patients, peripheral blood and intrahepatic MAIT cells **(A,B)** and peripheral blood and intrahepatic γδ T cells **(C,D)** were also quantified by flow cytometry revealing no increased infrequency of peripheral blood and intrahepatic MAIT cells or γδ T cells in AIH patients compared to healthy subjects or DILI patients.

Type I and type II NKT cells were quantified in peripheral blood and liver biopsies of AIH patients and control groups by flow cytometry. Exemplary stainings of mock-loaded human CD1d tetramer in comparison to sulfatide-loaded human CD1d tetramer are shown in Figure 1. Additionally, representative stainings comparing the frequency of peripheral blood and intrahepatic type I and type II NKT cells in a representative healthy subject and a representative AIH patient are presented in Figure 2. The frequency of peripheral blood sulfatide-reactive type II NKT cells was significantly increased in AIH patients (0.11% of peripheral blood leukocytes) in comparison to healthy subjects (0.05% of peripheral blood leukocytes) and DILI patients (0.06% of peripheral blood leukocytes, p < 0.01; Figure 3A). Furthermore, intrahepatic sulfatide-reactive type II NKT cells were significantly more frequent in AIH patients (3.78% of intrahepatic leukocytes) in comparison to healthy subjects (1.90% of intrahepatic leukocytes), and DILI patients (2.03% of intrahepatic leukocytes; *p* < 0.05; Figure 3B). We did not detect a significant difference in peripheral blood or intrahepatic type II NKT cell numbers between treatment-naive AIH patients with pronounced biological inflammatory activity (*n* = 5; 3.22% of intrahepatic leukocytes) and AIH patients in remission under immunosuppressive treatment (*n* = 7; 3.78% of intrahepatic leukocytes; not shown). In contrast to type II NKT cells, the frequency of peripheral blood and intrahepatic type I NKT cells was very low and did not differ significantly between patient groups (Figures 3C,D). Peripheral blood type II NKT cells mainly expressed CD4 (54.5%) or CD8 (36.8%), while type I NKT cells were predominantly double-negative (49.6%) or CD4+ (44.0%; Supplementary Figure S1). Only a minority of peripheral blood (median value 3.9%) or intrahepatic (median value 0.75%) sulfatide-reactive type II NKT cells expressed the γδ-T cell receptor (not shown). All peripheral blood and intrahepatic type I NKT cells were γδ-TCR negative, pursuant to their expression of the invariant Vα24-Jα18 TCR α-chain (not shown). To exclude that elevation of type II NKT cells in AIH patients was unspecific, peripheral blood and intrahepatic MAIT cells and γδ T cells, belonging also to the group of unconventional T cells like NKT cells, were quantified. Representative flow cytometric stainings of MAIT cells and γδ T cells are shown in Figure 4. The frequency of peripheral blood and intrahepatic MAIT cells and γδ T cells did not differ significantly between AIH patients and control groups (Figures 5A–D).

### Analysis of the Cytokine and Chemokine Receptor Profile of Peripheral Blood Type II NKT Cells in AIH Patients

To characterize peripheral blood sulfatide-reactive type II NKT cells, flow cytometric intracellular cytokine stainings were performed. Peripheral blood sulfatide-reactive type II NKT cells in AIH patients produced mainly TNFα (48.5% of type II NKT cells were TNFα+) and, to a lesser extent, IFNγ (8.6% of type II NKT cells were IFNγ+), IL-17 (0.6% of type II NKT cells were IL-17+) or IL-4 (3% of type II NKT cells were IL-4+; Supplementary Figures S2A–D). These results did not differ significantly from healthy subjects. In addition to the cytokine profile, chemokine receptor expression was analyzed which might help to identify the kind of intrahepatic antigen presenting cell (APC) that attracts type II NKT cells to the liver. Peripheral blood sulfatide-reactive type II NKT cells of AIH patients mainly expressed CXCR4 (41.7% of peripheral blood type II NKT cells), CXCR3 (32.2% of peripheral blood type II NKT cells), and CCR9 (28.2% of peripheral blood type II NKT cells), and less CXCR6 (3.6% of peripheral blood type II NKT cells; Supplementary Figure S3). These results did not differ significantly compared to control groups (not shown).

### Intrahepatic Sulfatide-Reactive Type II NKT Cells Show a Distinct Pro-inflammatory Cytokine Profile in Patients With Autoimmune Hepatitis

**Figure 6:**
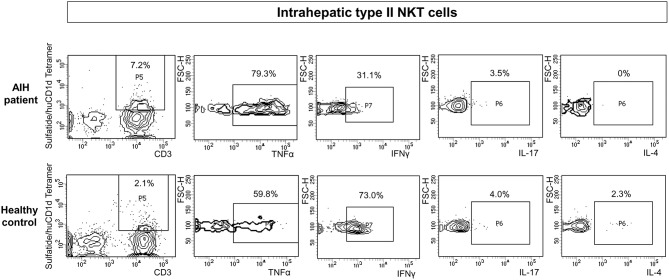
Exemplary flow cytometric intracellular cytokine stainings for type II NKT cells. Representative intracellular cytokine stainings for TNFα, IFNγ, IL-17 and IL-4 for intrahepatic type II NKT cells of AIH patients (upper row) and healthy subjects (lower row) are shown.

**Figure 7:**
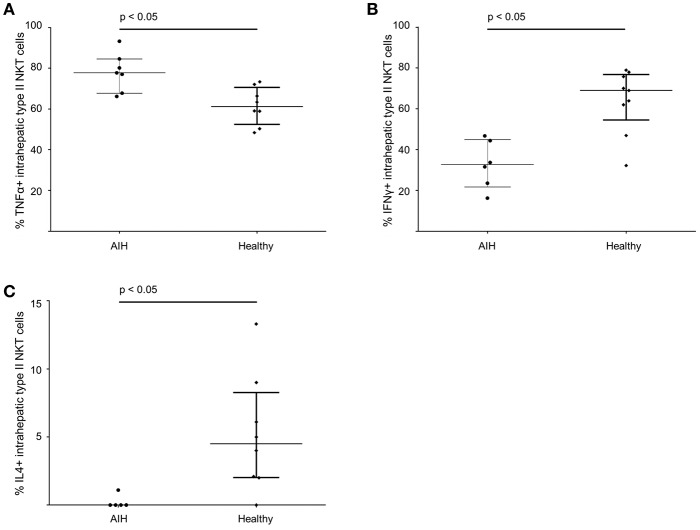
Intrahepatic sulfatide-reactive type II NKT cells in patients with autoimmune hepatitis reveal a different cytokine profile than in healthy subjects. Intrahepatic type II NKT cells of AIH patients have an increased expression of TNFα **(A)**, a lower expression of IFNγ **(B)** and show a complete lack of IL-4 **(C)** compared to healthy subjects.

The cytokine profile of peripheral blood sulfatide-reactive type II NKT might not reflect the local situation in the liver. Therefore, the cytokine profile of intrahepatic sulfatide-reactive type II NKT was analyzed (representative stainings are shown in Figure 6). In AIH patients, intrahepatic sulfatide-reactive type II NKT cells showed a differing cytokine profile in comparison to healthy subjects: TNFα expression was significantly higher in AIH patients compared to healthy subjects (77.8% of intrahepatic type II NKT cells were TNFα+ in AIH patients vs. 61.25% in healthy subjects; *p* < 0.05; Figure 7A), and IFNγ expression was significantly lower (32.7% of intrahepatic type II NKT cells were IFNγ+ in AIH patients vs. 69% in healthy subjects; *p* < 0.05; Figure 7B). Intrahepatic sulfatide-reactive type II NKT cells in AIH patients did not express IL-4 in contrast to healthy subjects (0 vs. 4.5% IL-4+ intrahepatic type II NKT cells; *p* < 0.05; Figure 7C). IL-17 expression of intrahepatic type II NKT cells in AIH patients did not differ significantly from healthy subjects (3.5 vs. 4.6%; *p* > 0.05; not shown). This cytokine profile of intrahepatic type II NKT cells in AIH patients was independent of the degree of liver inflammation quantified by mHAI (not shown).

### CD1d-Expression Is Elevated Mainly on Infiltrating CD4+ and CD8+ T Cells in Intrahepatic Portal Areas of AIH Patients

**Figure 8:**
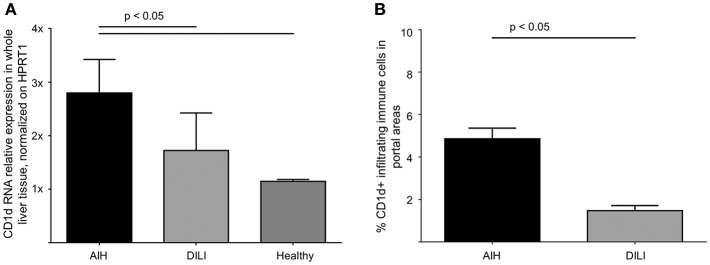
Intrahepatic expression of CD1d is elevated in patients with autoimmune hepatitis. Quantitative PCR analysis of relative RNA expression, normalized on HPRT1, in whole liver tissue samples revealed significantly elevated levels of CD1d RNA in AIH patients in comparison to DILI patients or healthy subjects **(A)**. Due to CD1d-RNA *in situ* hybridization, the expression of CD1d-RNA was significantly elevated in infiltrating lymphocytes in portal areas of patients AIH in comparison to patients with DILI **(B)**.

**Figure 9:**
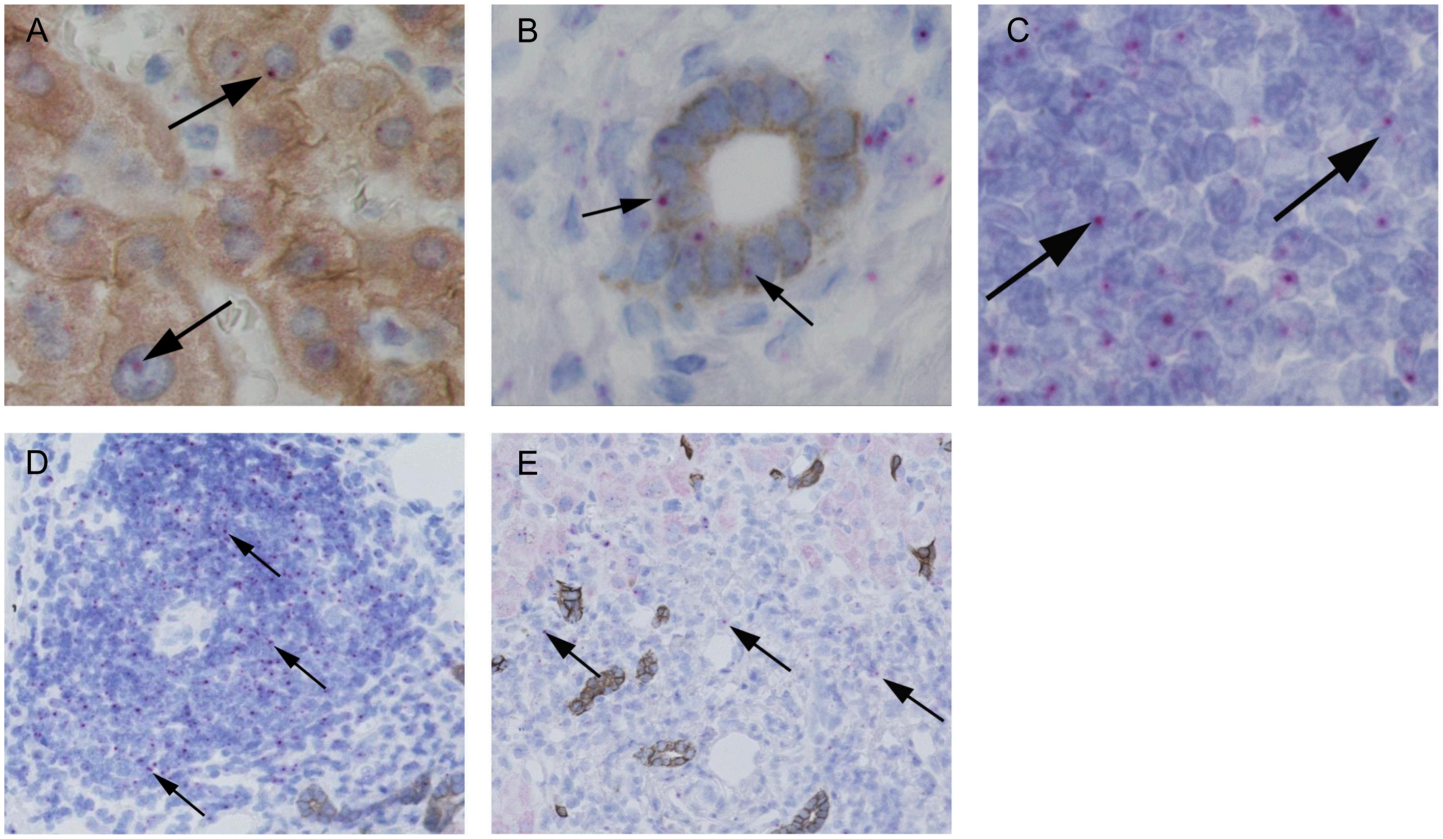
Representative *in situ* stainings of CD1d-RNA in liver biopsies. Representative stainings of CD1d-RNA (purple dots, see arrows) with co-stainings of hepatocytes (**A**, brown = CK18) and cholangiocytes (**B**, brown = CK7) and in infiltrating lymphocytes in portal areas **(C)**. Representative analyses of CD1d-RNA expression (see arrows) in infiltrating lymphocytes in portal areas of an AIH patient **(D)** and a patient with DILI **(E)**.

**Figure 10:**
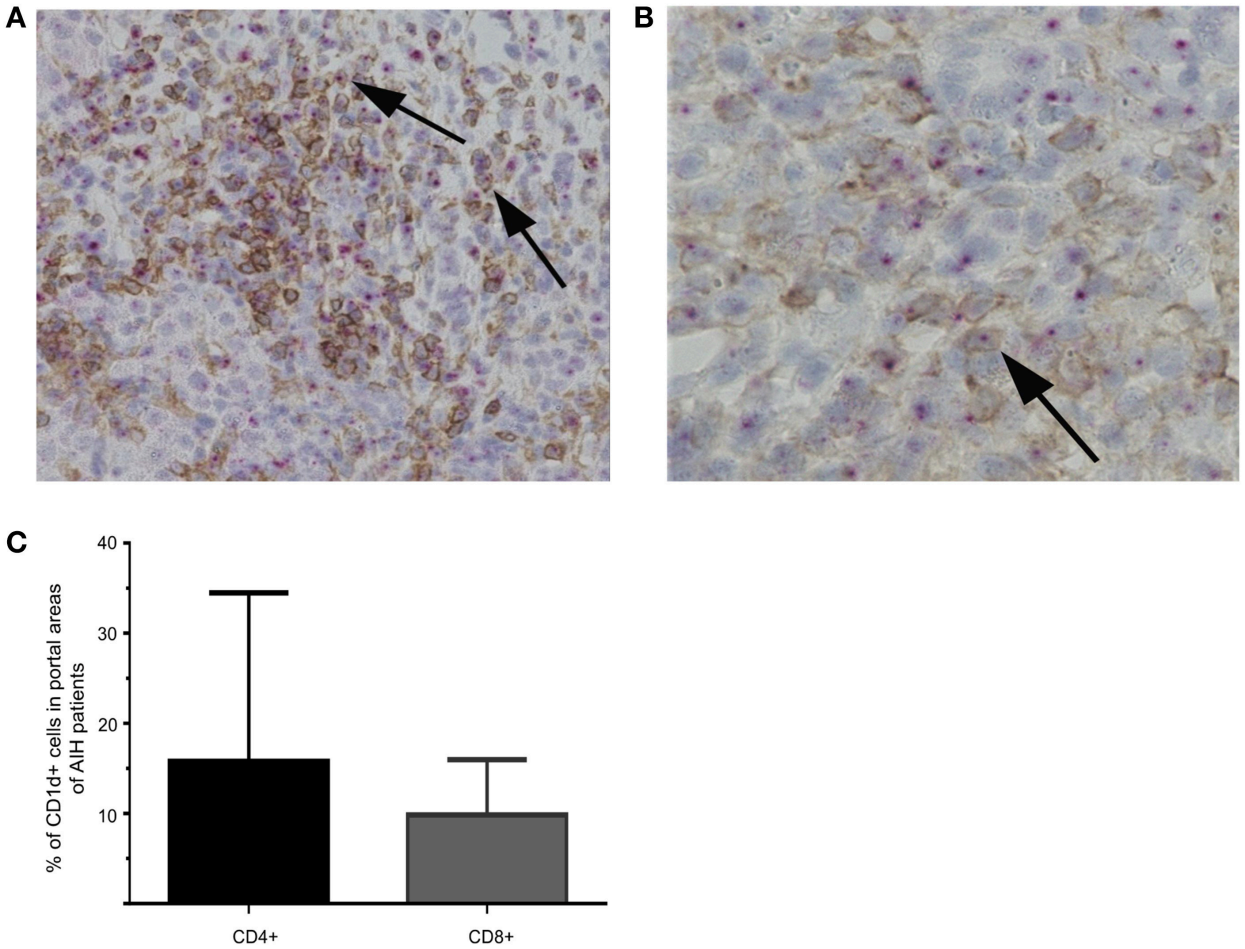
Co-stainings of CD1d-RNA with CD4 or CD8 in liver biopsies. Representative co-stainings of CD1d-RNA (purple dots) with CD4 (brown) in 20x **(A)** or 40x **(B)** magnification are shown. Double positive cells are marked by arrows. Percentage of CD4+CD1d (left bar) or CD8+CD1d (right bar) double positive cells in portal areas of AIH patients are displayed **(C)**.

The interaction of CD1d on APC and the TCR of NKT cells is highly specific for NKT cell lipid antigen recognition. Therefore, we investigated whether and on which cells CD1d expression is increased in livers of AIH patients. First, a quantitative PCR analysis of relative CD1d-RNA expression out of whole liver tissue samples of treatment-naive AIH patients in comparison to healthy liver tissue or patients with DILI was performed. The expression of CD1d RNA was significantly elevated in livers of AIH patients (2.8 fold expression, normalized to the expression of housekeeping gene HPRT1) as compared to healthy subjects (1.1 fold expression) or DILI patients (1.7 fold expression; Figure 8A). To identify the cellular source of CD1d expression, CD1d RNA was stained by *in situ* hybridization on histological slides of liver biopsies. Representative stainings of CD1d on hepatocytes, cholangiocytes or infiltrating immune cells in liver biopsies are shown in Figures 9A–C. Analysis of CD1d expression in portal and lobar intrahepatic areas revealed that CD1d was upregulated in portal areas with dense lymphocytic infiltrates in liver biopsies of AIH patients (Figure 9D). DILI patients had equivalent amounts of infiltrating immune cells in portal areas (Figure 9E) and equivalent levels of biochemical markers of liver inflammation and damage (median ALT 1301U/l, median total bilirubin 6.8 mg/dl) in comparison to AIH patients (median ALT 468U/l, median total bilirubin 3.3 mg/dl). Nonetheless, AIH patients showed a significantly elevated expression of CD1d on infiltrating immune cells in portal areas (4.9% of infiltrating immune cells were CD1d+) compared to DILI patients (1.5% of infiltrating immune cells were CD1d+, *p* < 0.05; Figure 8B). The median mHAI score of AIH patients for the analysis of CD1d expression was 7 out of 18. AIH patients did not show significant differences to the control group in their expression of CD1d on infiltrating immune cells in lobular areas, on cholangiocytes or on hepatocytes (not shown). Co-staining of CD1d with either CD4 (representative stainings are shown in Figures 10A,B) or CD8 revealed that infiltrating CD1d positive cells were mainly CD4+ T cells (up to 39.4% of CD1d+ portal cells were CD4+, median value 15.8%) and to lesser degree CD8+ T cells (up to 16.4% of CD1d+ portal cells were CD8+, median value 9.9%; Figure 10C).

## Discussion

Previous mouse studies about the role of NKT cell subtypes in immune-mediated liver diseases have reported a pro-inflammatory role of type I NKT cells and a more regulatory function of type II NKT cells (31). In mice, type II NKT prevent immune-mediated liver diseases by the induction of anergy in type I NKT cells or by inhibition of conventional T cells (5, 6, 22, 32). We here show that human intrahepatic type II NKT are increased and adopt a pro-inflammatory phenotype in AIH and could be activated by elevated CD1d-expression on infiltrating T cells. Importantly, these results are based on analyses out of human liver samples obtained at the time of active liver inflammation and not at end-stage liver disease.

In the literature, the frequencies of peripheral blood and intrahepatic type I and type II NKT cells vary between humans and mice. Type I NKT cells comprise about 0.2–0.5% of peripheral blood or spleen murine lymphocytes, but are enriched in livers and other organs and represent about 15–35% of liver lymphocytes in mice (12). In murine liver, type I NKT cells outnumber type II NKT cells (33). In humans, type II NKT cells (about 2% of peripheral blood lymphocytes) are more frequent in peripheral blood than type I NKT cells (0.01–0.5% of peripheral blood lymphocytes), consistent to our data (12, 34). In the past, the frequency of human intrahepatic NKT cells has been mainly analyzed by unspecific CD3/CD56 co-staining (“NKT-like” cells) or by staining of Vα24 to identify type I NKT cells. In one study, CD3+CD56+ “NKT-like” cells comprised 3–5% of liver mononuclear cells in livers of resected metastases of colonic carcinoma and from split liver grafts which served as healthy subjects (35). When type I NKT cell were defined by their expression of Vα24 and Vβ11 TCR-chains, they comprised 0.3%−0.5% of intrahepatic CD3+ cells in healthy livers (36). Taken together, these studies reveal a certain inconsistency regarding the numbers of intrahepatic NKT cells in healthy human liver and liver disease which is due to different staining approaches. The most specific stainings by αGalCer-loaded tetramers indicate that type I NKT are very rare in human livers, which contrasts with the frequency of murine intrahepatic type I NKT cells. In our study we show that the frequency of intrahepatic type I NKT cells is indeed very low comprising about 0.1% of intrahepatic leukocytes. However, we could show that type II NKT cells are more frequent in human livers. In healthy subjects, intrahepatic sulfatide-reactive type II NKT represent about 1.8% of intrahepatic leukocytes. In AIH, numbers of intrahepatic sulfatide-reactive type II NKT cells are increased and they represent about 3.8% of intrahepatic leukocytes. Apart from our group, others have also detected sulfatide-reactive type II NKT cells in human peripheral blood, intestinal mucosa and livers (37–39).

We here show that intrahepatic sulfatide-reactive type II NKT cells in AIH patients had upregulated TNFα- and downregulated IFNγ-expression. In comparison to healthy subjects, intrahepatic sulfatide-reactive type II NKT cells had completely suspended IL-4 production. This cytokine shift of sulfatide-reactive intrahepatic type II NKT cells, potentially favoring a classical Th1 response, seems to be part of pro-inflammatory mechanisms of AIH. Classically, the main pro-inflammatory drivers of AIH are considered to be CD4+ or CD8+ conventional T cells. However, since human NKT cells express CD3 and CD4 or CD8, NKT cells have probably been included in previous analyses and our results do not contradict former results. Indeed, from a quantitative perspective, conventional T cells still dominate the inflammatory infiltrates in AIH, but nevertheless, NKT cells seem to be an additional, potent source of cytokines. Future studies need to further define the role of intrahepatic NKT cells in human AIH: by rapid cytokine release, they could shape the immune response in AIH via interaction with different cell types, thus orchestrating other immune cells and their effector mechanisms. As an example, Sprengers et al. could show in a mouse model that type I NKT cells were able to stimulate conventional CD8+ T effector cells to an immune response against an antigen that was expressed in the liver (40). Furthermore, NKT cells could activate DC, Kupffer cells, and B cells via TCR-CD1d lipid-antigen interaction and additionally by CD40L-CD40-interaction (41). TNFα is a driving pro-inflammatory cytokine in AIH since anti-TNFα is a potent second-line therapy (42). TNFα could further promote the crosstalk between type II NKT cells and other intrahepatic immune cells such as γδ T cells and NK cells and their recruitment to the liver (43, 44). The role of IFNγ production by type II NKT cells is less clear.

In view of our analyses of the cytokine profile, we assume the role of intrahepatic type II NKT cells in AIH to be more pro-inflammatory, though the effects of the particular cytokines are probably more complex. Another well-established example for type II NKT cells adopting a more pro-inflammatory role is in inflammatory bowel disease, both in mouse models and in human ulcerative colitis (38, 45, 46). Interestingly, sulfatide-reactive IL-13Ralpha2+ type II NKT are enriched in the lamina propria of human ulcerative colitis and IL-13Ralpha2+ cells have also been recently identified in human liver (38, 39). In our analyses, type II NKT cell numbers and their cytokine profile were independent of whether AIH patients were treated immuno-suppressively or were treatment-naive. Though these subgroup analyses have to be considered with caution due to small patient numbers, this might reflect that the small, but elevated numbers of type II NKT cells are permanently present in AIH livers promoting its chronic and fluctuating course and attract larger numbers of conventional T cells in times of pro-inflammatory flares. Overall, the cytokine shift of intrahepatic type II NKT cells and their role for the chronic course of AIH deserves further investigations to address its potential therapeutic implication.

We show that CD1d-expression, which is essential for antigen presentation to both type I and type II NKT cells, is mainly upregulated on infiltrating immune cells in portal areas and not by cholangiocytes or hepatocytes in the livers of AIH patients. These CD1d positive, infiltrating immune cells are mainly CD4+ T cells and to lesser degree CD8+ T cells. However, a relevant proportion of CD1d positive cells in periportal infiltrations were neither CD4+ nor CD8+ T cells. In general, CD1d is expressed on APC such as dendritic cells (DC), macrophages, and B cells, whereas in murine livers, CD1d is also expressed on Kupffer cells and liver sinusoidal endothelial cells (LSEC) (41). CD1d is constitutively expressed on murine hepatocytes, but in humans the situation is less clear: Some authors have shown that CD1d-expression on hepatocytes is pronounced in end-stage liver disease, whereas others that CD1d is downregulated in cirrhotic livers (47, 48). CD1d expression on cholangiocytes has also been detected (48). This is in line with results showing that NKT cells convey bile duct inflammation in primary biliary cholangitis (PBC), next to AIH another autoimmune liver disease (49, 50). However, in our study CD1d expression was pronounced on infiltrating CD4+ T cells. This seems to be an AIH-specific effect since our control group with DILI patients had a comparable amount of portal immune cell infiltration but did not overexpressed CD1d. We cannot exclude that CD1d expression on B cells, DC, Kupffer cells or LSEC could also be relevant for the pathogenesis of AIH, but due to limited human material we were not able to test this.

Our results in human AIH are in line with a recent publication by Weng et al. which could show in a mouse model that the interaction between type II NKT cells and conventional T cells leads to a Th1-pronounced inflammatory milieu in the liver (23). Like in mouse models of inflammatory bowel disease, the pro-inflammatory switch of type II NKT cells was associated with a local overexpression of CD1d (45). Weng et al. could also show, that in liver samples of human AIH, CD1d is overexpressed on liver-infiltrating CD3+ immune cells in comparison to healthy subjects (23). Our study adds to these results by Weng et al. that upregulation of CD1d seems to be an AIH specific mechanism since our “inflammatory” control group consisting of DILI patients did not reveal such an upregulation. Furthermore, we have identified a pro-inflammatory phenotype of human type II NKT cells in human liver tissue of AIH, thus confirming the assumption of a pathogenic role for type II NKT cells in AIH by Weng et al. based on their results in a murine model of chronic autoimmune liver disease.

There are some limitations of our study, most of them are due to limited human material out of liver biopsies. For example, we could not perform cytokine analyses of intrahepatic type II NKT in DILI patients. Besides, we were not able to stain matched blood and liver samples from the same patients. Another aspect is choosing control groups for experimental analyses of human liver diseases, which is difficult and depends on the resources of the study center. We have chosen DILI as an inflammatory control group since, from a clinical point of view, this entity reaches a comparable level of acute intrahepatic inflammation like AIH. We did not identify a clinical parameter (such as grade of fibrosis, immunosuppressive treatment, age, sex or body mass index) correlating with intrahepatic type II NKT cell frequency or cytokine profile. Larger subgroup analyses are needed to address this issue.

In summary, in our study we show that intrahepatic sulfatide-reactive type II NKT cells are elevated in AIH patients and show a distinct cytokine profile with increased levels of TNFα, reduced levels of IFNγ and absence of IL-4. Besides, infiltrating immune cells in portal tracts, identified mainly as CD4+ and CD8+ T cells, overexpress CD1d in AIH which can activate type II NKT cells. Thus, type II NKT cells contribute to the pro-inflammatory mechanisms of AIH, contrasting their more regulatory role in mouse models, and are worthy of further studies, especially with regard to specific immune-modulating treatment of AIH patients.

## Data Availability

All datasets generated for this study are included in the manuscript and/or the Supplementary Files.

## Ethics Statement

This study was carried out in accordance with the recommendations of the local ethics committee Ethik-Kommission der Ärztekammer Hamburg, Weidestr. 122 b, 22083 Hamburg, Germany (PV3912, PV5184 and PV5187) with written informed consent from all subjects. All subjects gave written informed consent in accordance with the Declaration of Helsinki. The protocol was approved by the Ethik-Kommission der Ärztekammer Hamburg, Weidestr. 122 b, 22083 Hamburg, Germany.

## Author Contributions

MS performed the experimental analyses, interpreted data, and wrote the manuscript. JW and PF performed experimental analyses. LF enabled the acquisition of healthy liver tissue from the resection margin of liver adenomas. SW and TK provided the histological stainings of liver biopsies. CW-N, MP, and JHa recruited patients into the study. ET, JHe, CS, and AL advised on experimental design, reviewed and edited the manuscript. PA devised and designed experiments, interpreted data, reviewed and edited the manuscript.

### Conflict of Interest Statement

The authors declare that the research was conducted in the absence of any commercial or financial relationships that could be construed as a potential conflict of interest.
