# Supplementary material for: Inflammatory Phenotype of Intrahepatic Sulfatide-Reactive Type II NKT Cells in Humans With Autoimmune Hepatitis
Source: Front Immunol. 2019 May 28;10:1065. doi: 10.3389/fimmu.2019.01065 (PMC6546815; doi:10.3389/fimmu.2019.01065)
Supplement: Supplementary file 1 [file Table_1.docx]

Supplementary Material

**METHODS**

**Clinical characteristics of patients and control groups**

General clinical characteristics of patients and controls that served for analyses out of peripheral blood are displayed in table 1. Of all AIH patients that served for peripheral blood analyses, 20 were ANA and anti-SMA double-positive and 13 were ANA single-positive. Eight AIH patients were anti-SLA/LP positive and three were anti-LKM positive. Median titers of ANA and anti-SMA were 1:640, respectively. Of those AIH patients receiving immunosuppressive treatment (n=33), most patients received a combination of prednisolone and azathioprine (n=14) or a monotherapy of azathioprine (n=8). The median milligram dosage per kilogram body weight was 0.1 (0.1-0.9) for prednisolone and 1.5 (0.3-2.2) for azathioprine. Six AIH patients were treated with budesonide, two with mycophenolate mofetil, two with mercaptopurine and another with tacrolimus. Of those AIH patients under therapy, 15 patients showed biochemical activity of AIH defined as elevated transaminases and/or elevated gammaglobulins / IgG.

Clinical characteristics of those patients that served for analyses of liver biopsies are displayed in table 2. Of all AIH patients with liver biopsies (n=28), 11 were ANA and anti-SMA double-positive and nine were ANA single-positive. Two were anti-SLA/LP positive and one anti-LKM positive. The median titer of ANA was 1:1000 and the median titer for anti-SMA was 1:640. Of those AIH patients receiving immunosuppressive treatment (n=12), most patients received azathioprine (n=4) or prednisolone (n=4) monotherapy; two patients were under combination therapy of prednisolone and azathioprine. The median dosage of prednisolone was 0.18mg per kilogram body weight and the median dose of azathioprine was 1.6mg per kilogram body weight. One patient was on budesonide and another patient on mycophenolate mofetil. Of those AIH patients under therapy that had a liver biopsy, all 28 patients showed biochemical activity of AIH defined as elevated transaminases and/or elevated gammaglobulins / IgG.

The 10 DILI patients for peripheral analyses (table 1) and those 11 for intrahepatic analyses (table 2) were all of idiosyncratic pathogeneses with no patients suffering from acetaminophen-induced DILI. All DILI-patients showed a hepatocellular biochemical pattern with pronounced elevation of transaminases. Most of the DILI cases were due to antibiotics or phenprocoumon.

**Flow cytometry of peripheral blood or intrahepatic unconventional T cells**

For the loading of CD1d-tetramers with αGalCer or sulfatide and for the staining of NKT cells with these lipid-loaded tetramers, titration experiments were performed. The following molar ratios and concentrations have been identified to be optimal and are in line with previous publications: Human PE-labelled CD1d-tetramers (NIH Tetramer Core Facility, Atlanta, Georgia, USA) were loaded with either αGalCer (‘synthetic’, α-Galactosyl Ceramide, Avanti Polar Lipids, Alabaster, Alabama, USA) at a molar ratio of 1:9 or sulfatide (‘bulk’ sulfatides of bovine origin, Sulfatides, Matreya, State College, Pennsylvania, USA) at a molar ratio of 1:3 for 18-20 hours. Before loading of CD1d tetramers, all lipids were dissolved in vehicle (0.5% polysorbate-20 /0.9% NaCl solution; Polysorbate-20 (TWEEN-20, Sigma, Steinheim, Germany). For the staining of type I or type II NKT cells, CD1d-tetramers loaded with αGalCer or sulfatide were used at a concentration of 1pmol/μl or 20pmol/μl, respectively. As a negative control, we used mock-loaded human CD1d-tetramers. CD19 positive cells were excluded to prevent unspecific tetramer-staining. Before staining of unconventional T cells in blood and liver samples with fluorochrome-labelled antibodies or lipid-loaded CD1d tetramers, PBMC and intrahepatic immune cells were pre-incubated on ice with an Fc receptor binding inhibitor (Fc Receptor Binding Inhibitor Polyclonal Antibody, eBioscience, Frankfurt, Germany).

Type I NKT cells were defined as CD3 positive, αGalCer-loaded human CD1d tetramer positive cells. Type II NKT cells were defined as CD3 positive, sulfatide-loaded human CD1d tetramer positive cells. MAIT cells were detected by staining of CD161 high and Vα7.2, the α-chain segment of their semi-invariant T cell receptor. An antibody against the gamma- and delta chains of the TCR in combination with CD3 was used to stain γδ T cells.

All given percentages of peripheral blood type I and type II NKT cells, MAIT cells and γδ T cells were referred to total peripheral blood leucocyte numbers. Thereby, we excluded a bias of our results in patients that might have lower numbers of leucocytes due to immunosuppressive treatment.

For cytokine analyses, peripheral blood or intrahepatic cells were stimulated with PMA (Phorbol-12-myristat-13-acetat, 5ng/ml, Sigma-Aldrich, Steinheim, Germany) and ionomycin (Ionomycin calcium salt, 1µg/ml, Sigma-Aldrich, Steinheim, Germany) in the presence of a protein transport inhibitor (BD GolgiPlug™, 1µl/ml, BD Biosciences, Heidelberg, Germany) in medium (RPMI Medium 1640, 1x; Gibco, Darmstadt, Germany) supplemented with Penicillin/ Streptomycin and 5 % fetal calf serum (FCS) for 4 hours at 37°C in a dark incubator with 5% CO2. After washing, the staining of vital cells and extracellular markers was performed before permeabilization and intracellular staining of cytokines (IntraStain, Dako, Hamburg, Germany). Chemokine receptors were stained in combination with extracellular markers.

**FIGURES**

**Fig. S1 Distribution of CD4 and CD8 expression on peripheral blood type II and type I NKT cells**

Peripheral blood type II NKT cells (left) and peripheral blood type I NKT (right) cells were analyzed whether they express CD4 or CD8 or are double negative (DN) for both CD4 and CD8.

**Fig. S2 Cytokine profile of peripheral blood sulfatide-reactive type II NKT cells of patients with autoimmune hepatitis**

The frequency of TNFα+ (A), IFNγ+ (B), interleukin-(IL-)17+ (C) and IL-4+ (D) peripheral blood type II NKT cells in AIH patients in comparison to healthy subjects is shown.

**Fig. S3 Chemokine receptor profile of peripheral blood sulfatide-reactive type II NKT cells of patients with autoimmune hepatitis**

The expression of the chemokine receptors CXCR3, CXCR4, CXCR6 and CCR6 was analyzed on peripheral blood type II NKT cells of AIH patients.
