# Supplementary figures and images for: Inflammatory Phenotype of Intrahepatic Sulfatide-Reactive Type II NKT Cells in Humans With Autoimmune Hepatitis
Source: Front Immunol. 2019 May 28;10:1065. doi: 10.3389/fimmu.2019.01065 (PMC6546815; doi:10.3389/fimmu.2019.01065)

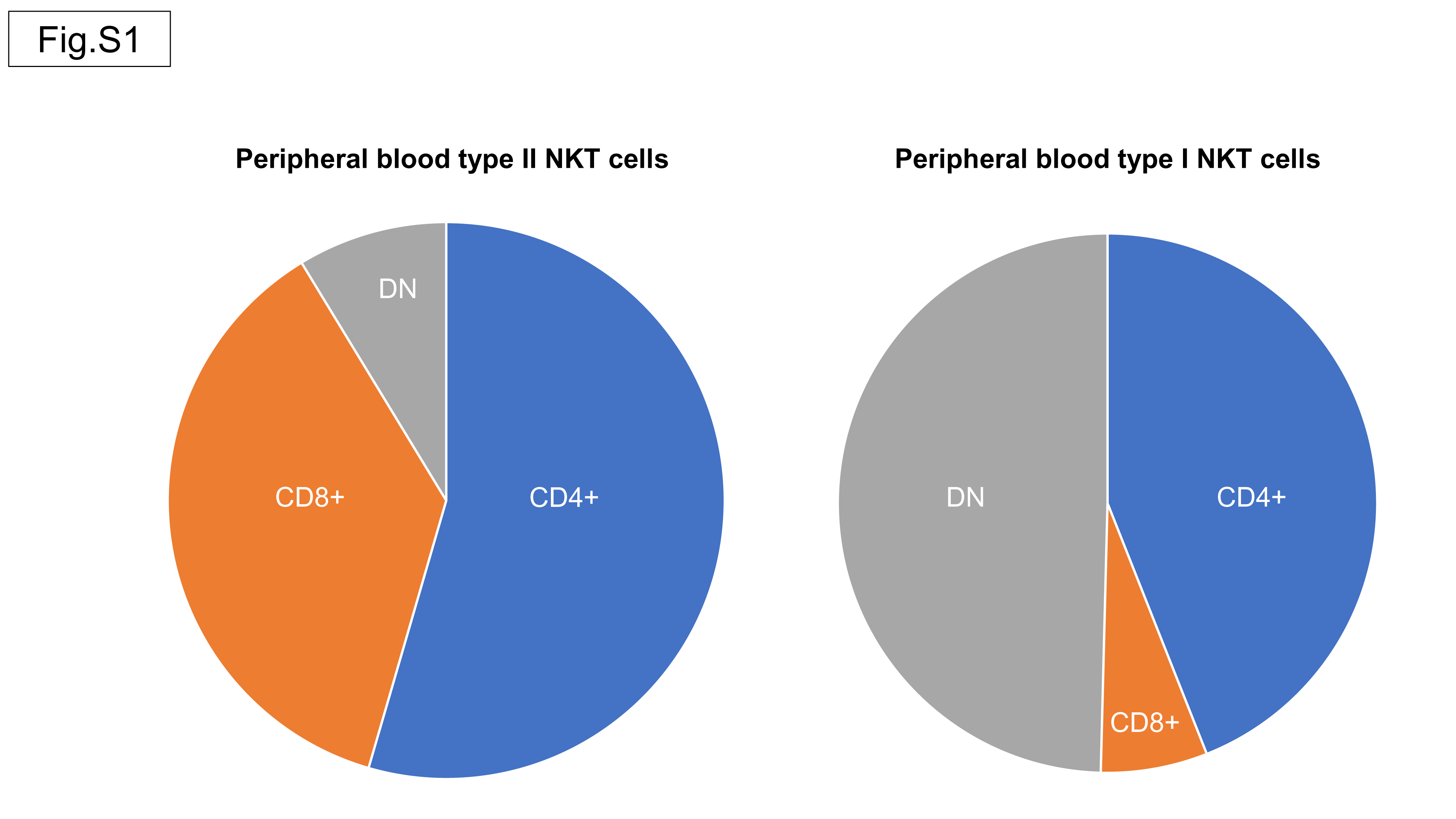

Supplement: Supplementary file 2 [file Image_1.TIF]

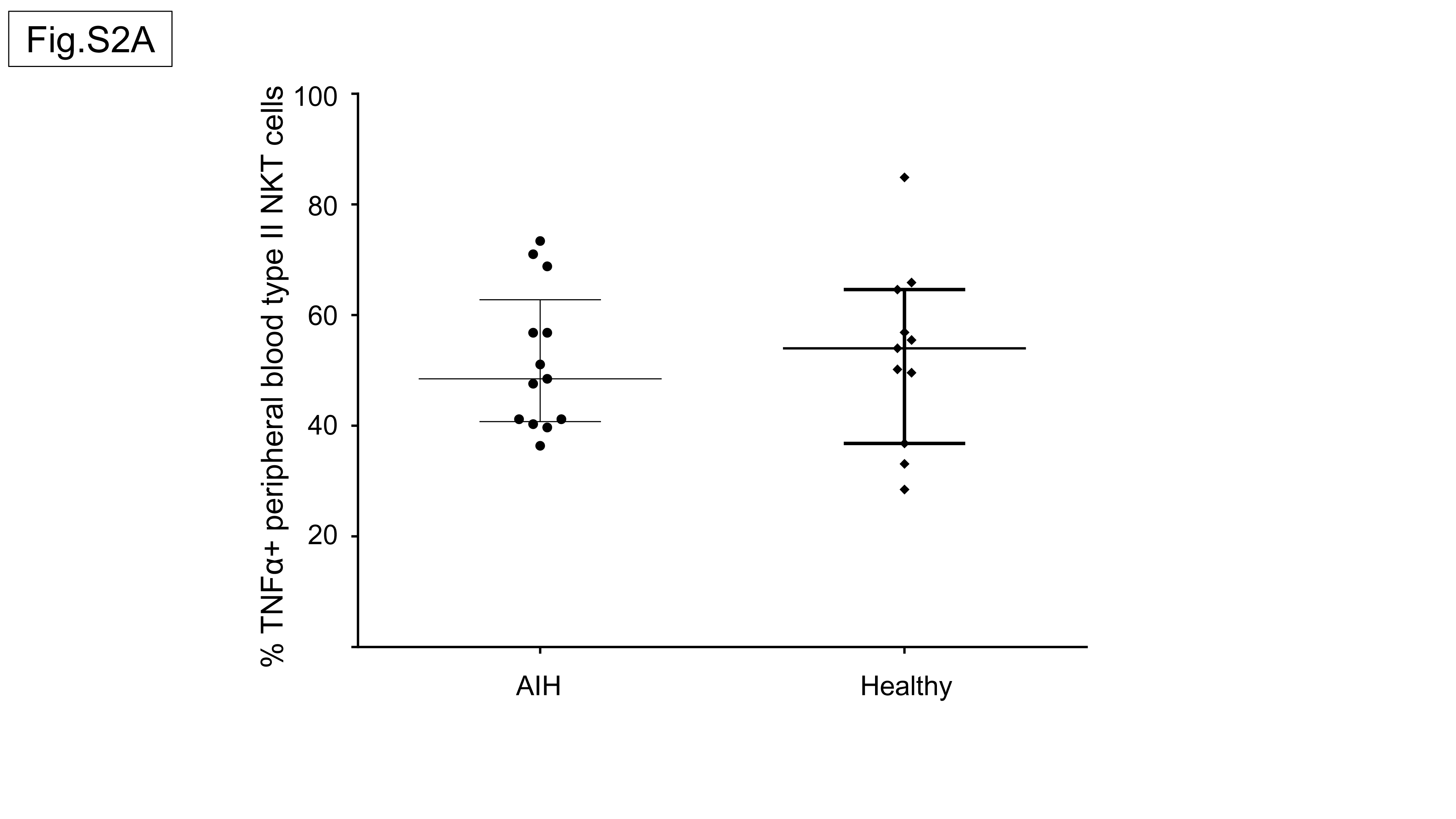

Supplement: Supplementary file 3 [file Image_2.TIF]

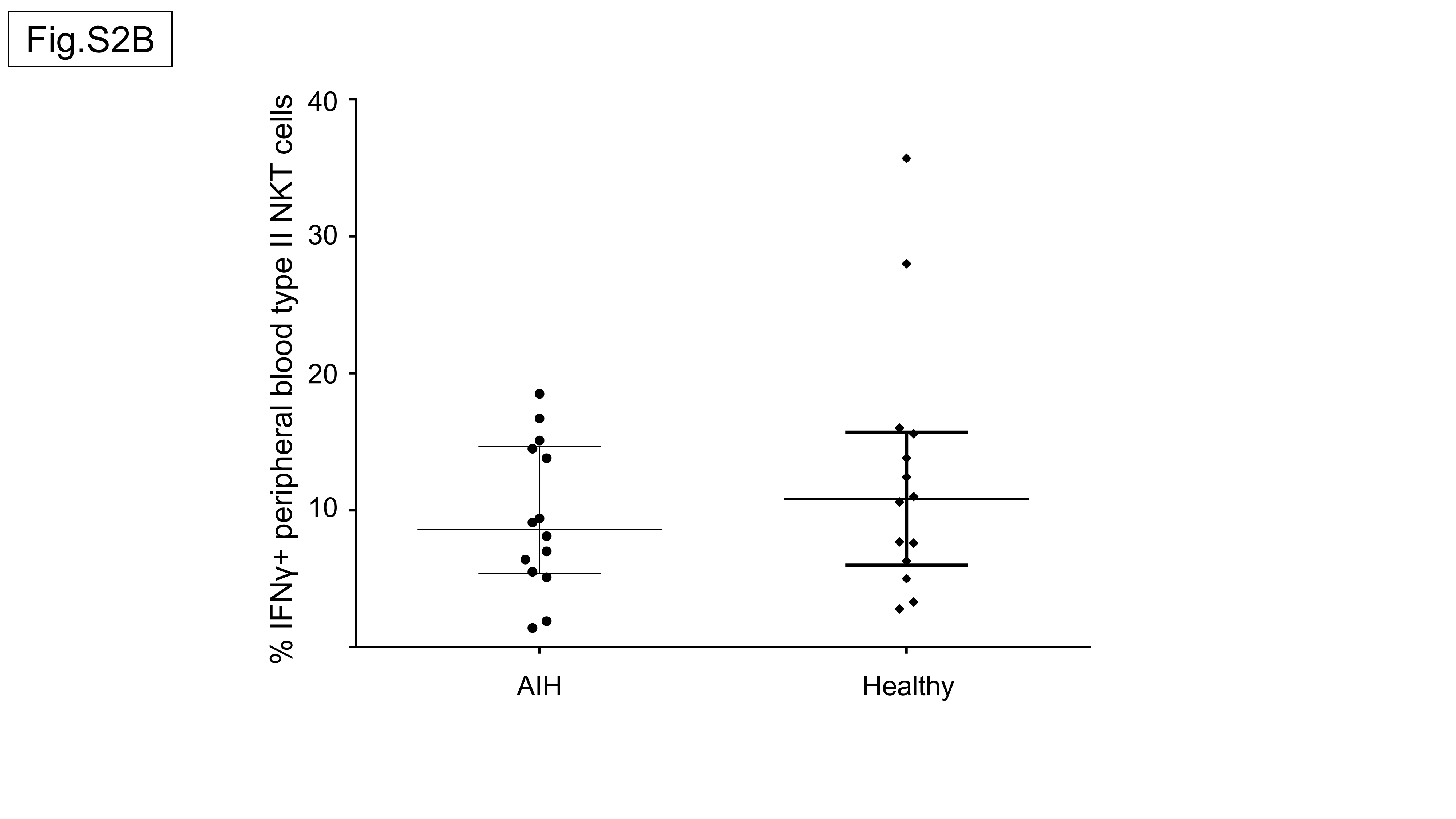

Supplement: Supplementary file 4 [file Image_3.TIF]

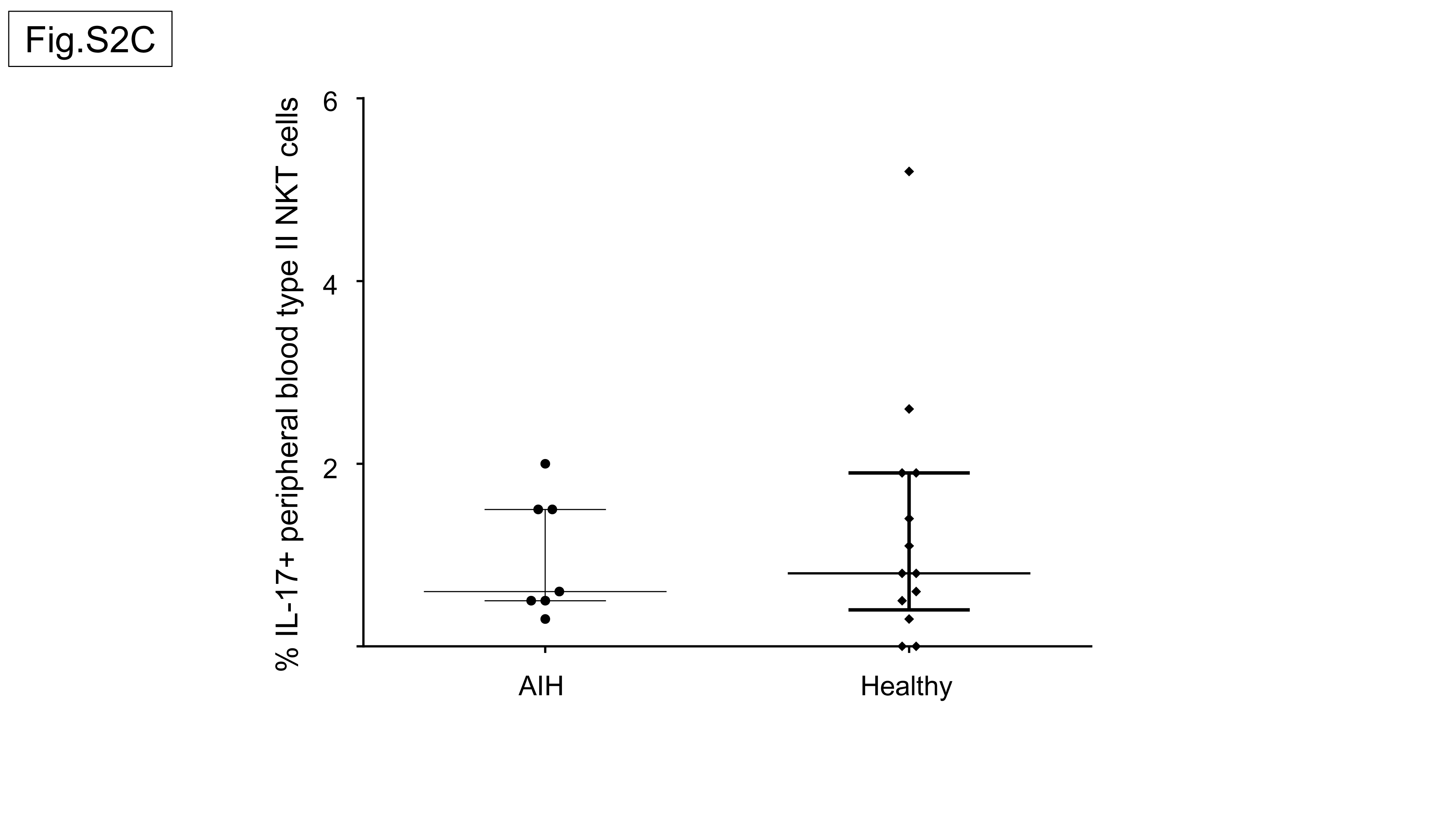

Supplement: Supplementary file 5 [file Image_4.TIF]

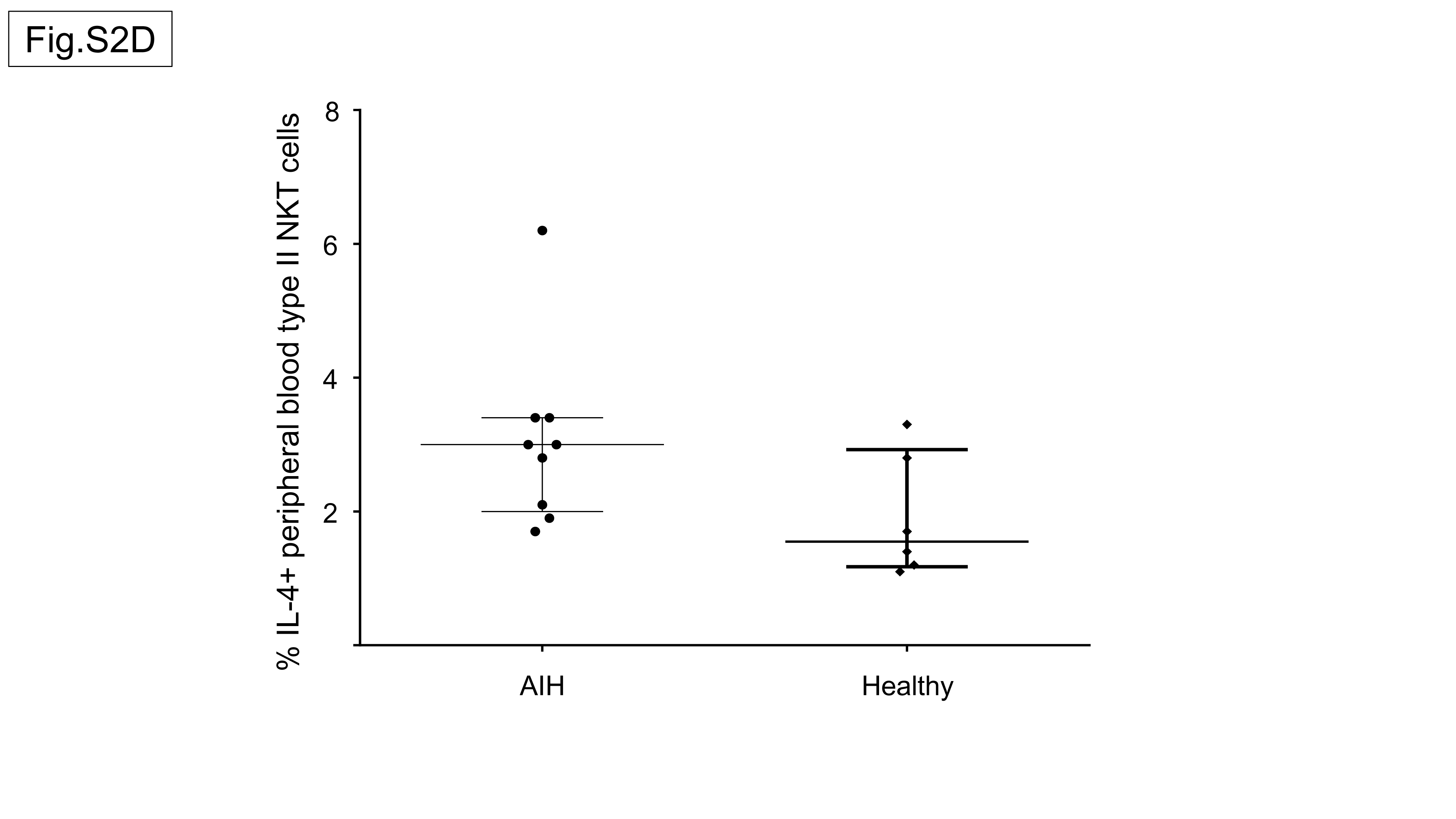

Supplement: Supplementary file 6 [file Image_5.TIF]

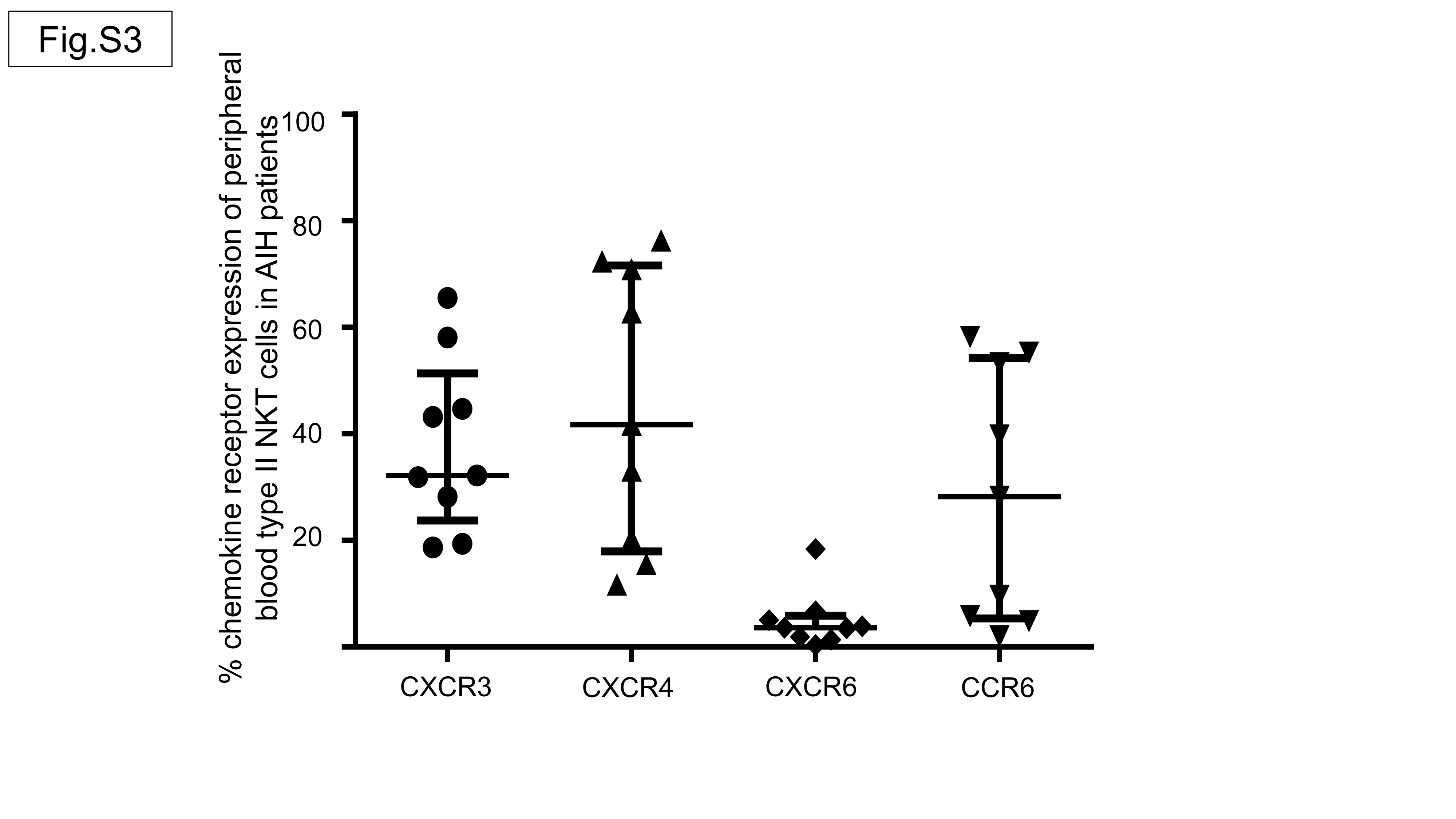

Supplement: Supplementary file 7 [file Image_6.TIF]
